# Teenage Male with Cough and Recurrent Bruit

**DOI:** 10.1155/2021/9453574

**Published:** 2021-11-22

**Authors:** Sheema Gaffar, Elliot Tucker

**Affiliations:** ^1^Department of Pediatrics, Eastern Virginia Medical School, 700 West Olney Road, Norfolk, VA 23507, USA; ^2^Children's Hospital of The King's Daughters, 601 Children's Lane, Norfolk, VA 23507, USA; ^3^Neonatal-Perinatal Medicine Fellowship Program, LAC+USC/CHLA, Keck School of Medicine at University of Southern California, Los Angeles, CA 90033, USA; ^4^Children's Specialty Group, Pediatric Cardiology, 601 Children's Lane, Norfolk, VA 23507, USA

## Abstract

A general pediatrician is skilled at continuity; through longitudinal evaluation, they serve as front-line providers in the recognition and referral of unusual pathology. The majority of arteriovenous malformations (AVM) are diagnosed with history and physical examination. AVM are inherently progressive by nature; their *expansion* is what creates the risk of morbidity. With higher-risk vascular lesions, *relative risk* is important when discussing management with observation versus with invasive intervention. Size, location, and expected course of progression of the lesion help generate a timeline for action. Collaboration of physicians with diverse expertise generates optimal plan of therapy, particularly when faced with an unusual clinical finding. Genetics referral may be beneficial, as the body of literature on AVM is growing, and databases on associated syndromes are evolving. Establishing concrete follow-up is imperative to assess for recurrence of AVM or development of additional symptoms. This can be with the interventionalist or with the generalist.

## 1. Case Presentation

**Figure 1:**
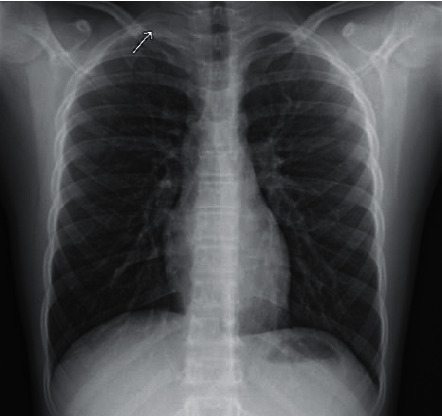
Chest radiograph showing a rounded density (32 mm × 22 mm) at the right lung apex.

An 11-year-old male was noted to have a bruit on exam and was referred to Cardiology department for evaluation. Workup included normal echocardiogram and vascular ultrasound of the neck arteries bilaterally, and he was managed expectantly. Three years later, he presented to his pediatrician with dry cough, nasal congestion, and sore throat of 5 days' duration. He was afebrile and well-appearing. Symptoms did not improve with supportive care, so he was prescribed a course of antibiotics. He gradually improved; however, his cough persisted and became more stridulous in nature. One week later, he again presented to his pediatrician with persistent cough. Neck radiographs obtained at that time showed normal airway anatomy, with an incidental “rounded contour” measuring 32 mm by 22 mm in the apex of the right lung (Figure 1). Follow-up chest radiograph noted that this rounded density was similar in appearance to supraclavicular lymph nodes, chest wall mass, mass of neural origin, or abscess. Clinically, the patient continued to be afebrile, with normal vital signs, negative rapid strep test, and no known infectious exposures. He had an interim vocal cord evaluation with otolaryngology that was unremarkable.

On physical exam, this is a well-developed adolescent male at the 60th percentile for height and weight, with normal vital signs. Auscultation revealed a right neck bruit and palpable thrill, with no pulsatile mass and no dermal discoloration. Remainder of the physical exam was normal. His past medical history includes bedwetting, irritable bowel syndrome, sleep difficulties, autosomal dominant polycystic liver disease, and congenital renal cysts that were reportedly not polycystic kidney disease. He has undergone colonoscopy, esophagoduodenoscopy, and anorectal manometry in the past as well. Family history is significant for high blood pressure, depression, cancer, inflammatory bowel disease, and insulin-dependent diabetes.

## 2. Diagnosis

**Figure 2:**
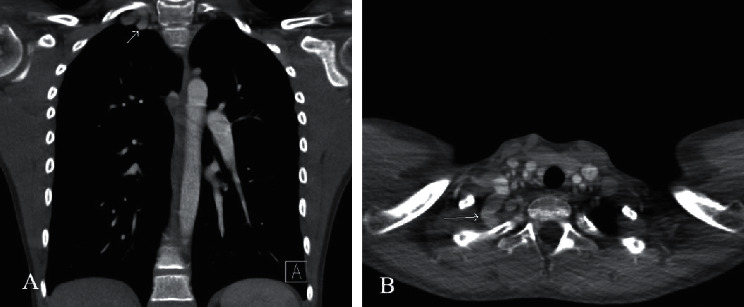
Computed tomography of chest with contrast; coronal (a) view and transverse (b) thin-slice view of the aberrant blood vessel arising from the right brachiocephalic artery, between the right vertebral and right subclavian arteries.

**Figure 3:**
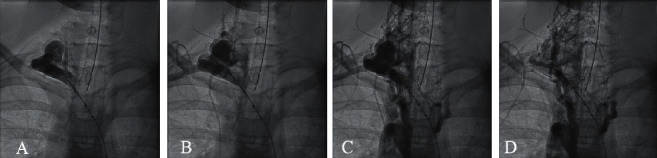
Serial images from right subclavian arteriogram show initial arterial phase (a, b) with subsequent venous phase of AVM (c, d).

**Figure 4:**
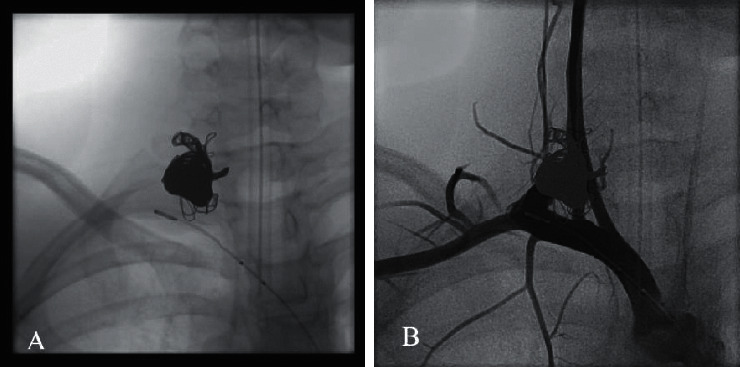
Images from the conclusion of the case demonstrating the mass of coils after occlusion (a) and filling of only normal arterial structures (b) on the postintervention angiogram.

Computed tomography imaging of his chest showed a substantial, tortuous blood vessel originating from the right brachiocephalic artery, with multiple branches, and prominent associated veins (Figure 2). Aortic arch, superior vena cava, cardiac anatomy, and airway anatomy were all normal. Coronaries were not fully visualized but appeared grossly normal, and incidental multiple renal cysts were noted. Overall impression was suggestive of arteriovenous fistula. The patient was referred to Interventional Pediatric Cardiology at that point. Due to variations in thyrocervical trunk anatomy, a co-operative diagnostic angiography was performed with both interventional cardiology and interventional neuroradiology demonstrating no important spinal or thyroid blood supply arising from the arteriovenous malformation (AVM) (Figure 3). Given the natural history that this AVM is likely to continue to expand over time and would be amenable to occlusion, the patient underwent successful transcatheter occlusion with 17 large detachable coils (Figure 4).

Initially, the differential for this unilateral apical mass on chest radiograph included vascular structure, lymph node, or intraparenchymal lesion. The concomitant upper respiratory symptoms suggested enlarged supraclavicular lymph node as the most plausible etiology. However, its vascular nature became apparent with further characterization on chest CT, alluding to the possibility of AVM or arterial dissection with pseudoaneurysm. Subclavian steal steno-occlusive disease or abnormal aortic arch was distant on the differential, as the patient had a normal left-aortic arch and normal vital signs with no hemodynamic instability at any point in presentation or workup.

## 3. Discussion

Primary AVM are rare. Arteriovenous fistulas are described in the adult literature, mostly in the context of iatrogenic fistulas created for dialysis access. These are even more rarely reported in pediatrics, particularly when not associated with a genetic syndrome such as collagen vascular disorder. The lack of literature specifically on primary AVM precludes research-based treatments. Instead, physicians use clinical judgement, guidelines compiled from adult studies, and disparate pediatric cases to create a treatment algorithm. The AVM's expansion or *potential* to expand is the primary cause of morbidity [1]. Since progression was expected, elective, nonurgent intervention was recommended and performed.

Our experience with an unusual finding in the presence of a physical exam finding that is usually innocent in children highlights the importance of the general pediatrician's role in longitudinal reevaluation and recognition and referral of unusual pathology. Ninety percent of AVM are diagnosed by history and physical exam [1, 2], and literature approximates that three-fourths of patients with AVM will require treatment in childhood or adolescence. Due to the dynamic nature of these lesions, it is wise to refer to a specialist for further evaluation and possible therapy. In addition, considering genetics referral may be beneficial when there are other medical problems, as in this patient, who had incidental renal cysts.

While recovery is largely subjective in terms of symptom abatement, a more objective assessment of therapeutic efficacy can be made in follow-up. By establishing routine postprocedural imaging or concrete intervals for surveillance [3, 5], a physician can evaluate if the intervention was successful in controlling the AVM's expansion.

## 4. Patient Course

This patient tolerated transcatheter occlusion of the arteriovenous fistula well and recovered as expected. He was discharged on postprocedure day 2, with Cardiology follow-up one month later. Other than achy right-sided shoulder pain over the first 1-2 weeks postocclusion, the patient had no issues after the procedure. Repeat imaging has not been performed to date in this patient as he has had no recurrence of his bruit in almost 3 years.

## Data Availability

No data were used to support this study.
